# Postnatal development of cerebrovascular structure and the neurogliovascular unit

**DOI:** 10.1002/wdev.363

**Published:** 2019-10-01

**Authors:** Vanessa Coelho‐Santos, Andy Y. Shih

**Affiliations:** ^1^ Center for Developmental Biology and Regenerative Medicine Seattle Children's Research Institute Seattle Washington; ^2^ Department of Pediatrics University of Washington Seattle Washington

**Keywords:** capillary, endothelial cell, pericyte, two‐photon imaging, vascular

## Abstract

The unceasing metabolic demands of brain function are supported by an intricate three‐dimensional network of arterioles, capillaries, and venules, designed to effectively distribute blood to all neurons and to provide shelter from harmful molecules in the blood. The development and maturation of this microvasculature involves a complex interplay between endothelial cells with nearly all other brain cell types (pericytes, astrocytes, microglia, and neurons), orchestrated throughout embryogenesis and the first few weeks after birth in mice. Both the expansion and regression of vascular networks occur during the postnatal period of cerebrovascular remodeling. Pial vascular networks on the brain surface are dense at birth and are then selectively pruned during the postnatal period, with the most dramatic changes occurring in the pial venular network. This is contrasted to an expansion of subsurface capillary networks through the induction of angiogenesis. Concurrent with changes in vascular structure, the integration and cross talk of neurovascular cells lead to establishment of blood–brain barrier integrity and neurovascular coupling to ensure precise control of macromolecular passage and metabolic supply. While we still possess a limited understanding of the rules that control cerebrovascular development, we can begin to assemble a view of how this complex process evolves, as well as identify gaps in knowledge for the next steps of research.

This article is categorized under:

Nervous System Development > Vertebrates: Regional Development
Vertebrate Organogenesis > Musculoskeletal and Vascular
Nervous System Development > Vertebrates: General Principles

## Graphical abstract

Assembly of the Neurogliovascular Unit during Development.

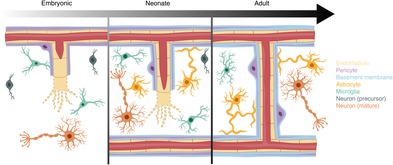

## 1 INTRODUCTION

The brain represents only ∼2% of the body's total mass, yet consumes ∼20% of its resting energy production. This corresponds to a 10‐fold higher rate of energy consumption, compared to an equivalent volume of tissue in the rest of the body (Mink, Blumenschine, & Adams, 1981). The high metabolic demand of the brain is fuelled by a constant flow of blood, supplied through a pervasive network of small arterioles, capillaries, and venules. The brain's microvasculature is remarkable in several ways. First, it is densely packed into brain tissue. Every cubic millimeter of brain tissue contains a meter of total vascular length (Tsai et al., 2009). Extrapolated over the entire brain, this means that ~400 miles of vasculature is held within the adult human brain, most of which is composed of miniscule capillaries. Second, the microvasculature in the cerebral cortex is organized such that every cell is no more than 15 μm away from a capillary (Blinder et al., 2013). This ensures that every brain cell is adequately supplied with oxygen and nutrients, including those distant from an arteriole perfusion source. Third, the brain vasculature is unique in its ability to form a blood–brain barrier (BBB), which tightly regulates the passage of molecules in and out of the brain. These barrier properties are first established during embryogenesis, but continue to be refined after birth. Fourth, the brain is a highly dynamic organ with fluctuating energy demands. The microvasculature must also be able to communicate with neurons to dilate or constrict during the regulation of cerebral blood supply.

There is still much to be learned about how the cerebrovasculature develops to become an efficient system for blood supply. However, existing data have already revealed an evolving growth and refinement in microvascular structure, which is contrasted to the marked stability of vascular networks of the adult brain (Cudmore, Dougherty, & Linden, 2017; Drew et al., 2010; Harb, Whiteus, Freitas, & Grutzendler, 2012). This process involves interplay between endothelial cells and mural cells (smooth muscle cells and pericytes) as chief engineers of the vessel wall, and then the gradual incorporation of other brain cell types (glia and neurons) and structural components (basement membrane) to further specialize vascular structure and function. In this review, we will explore the process of cerebrovascular development with a primary focus on changes in vascular network structure in the initial month after birth, primarily in rodent models. To appreciate the end product, we first present the microvasculature of the adult mouse cerebral cortex, which is a well‐studied three‐dimensional (3D) network with distinct microvascular zones. We then detail the establishment of this network during postnatal development and how it gains its key physiological roles, with a primary focus on BBB integrity. We further describe endothelial interactions with other cell types to support the sculpting and refinement of the capillary network. Finally, we discuss how live imaging could help grow our knowledge on postnatal vascular development. This review draws primarily on data from rodent cerebral cortex, and thus timing of events in other central nervous system regions, and other model organisms, may differ.

## 2 THE DESIGN OF A MATURE BRAIN MICROVASCULAR NETWORK

**Figure 1:**
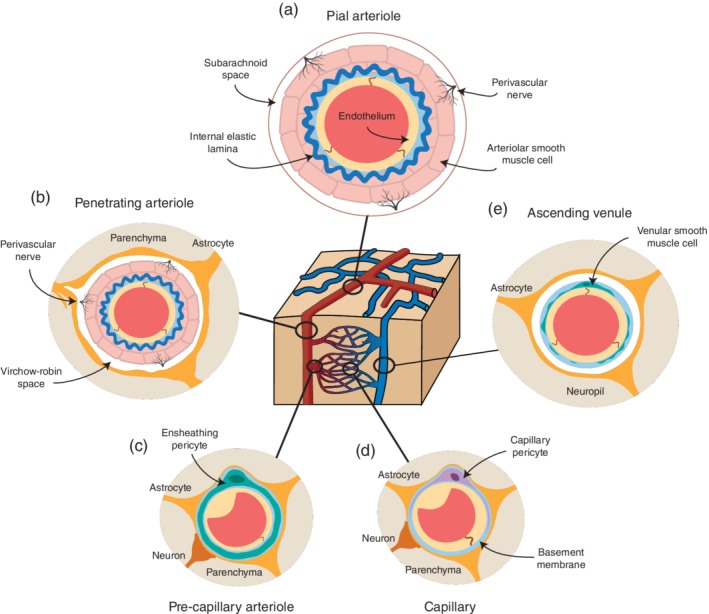
Neurovascular unit of the adult rodent brain. The cellular components of the vascular wall across different microvascular zones in cerebral cortex. This schematic shows cross‐sectional views of the vascular wall composition at the level of pial arterioles on the brain surface (a), and downstream penetrating arterioles (b), precapillary arterioles (c), capillaries (d), and ascending venules (e) within the brain parenchyma

To illustrate the structural features of brain microvasculature, we turn to the adult cerebral cortex. At the brain surface, pial arterioles lie within the subarachnoid space of the brain meninges (Figure 1a). These arterioles range from tens to hundreds of micrometers in diameter and are composed of an endothelial layer and internal elastic lamina, surrounded by 2–3 cell layers of concentric, ring‐link contractile smooth muscle cells. These arterioles also have an outer adventitial layer composed mostly of collagen fibers and perivascular nerves. This wall composition enables arterioles to dilate or constrict under the control of peripheral nerve ganglia or intrinsic brain neurons, which enables to modulation of cerebrovascular tone and brain perfusion (Hamel, 2006).

BOX 1 **RELATING ANIMAL MODELS TO HUMAN DEVELOPMENT**

Early‐life development is characterized by dramatic changes that can impact brain function over an entire lifespan. Like with rodents, a considerable portion of human brain development occurs after birth, with individual regions of the brain maturing at different rates (Norman & O'Kusky, 1986). Nevertheless, cross‐comparisons of developmental benchmarks between rodents and humans, including the timing of neuroanatomical changes, neurogenesis, synaptogenesis, gliogenesis, and myelination, have revealed that the rodent brain at P1 to P5 roughly corresponds roughly to 23–32‐weeks of gestation in human development (Semple, Blomgren, Gimlin, Ferriero, & Noble‐Haeusslein, 2013). P10 in rodents roughly corresponds to 40‐weeks of gestation in human development. Thus, studying rodent models just within the first 2‐weeks following birth provides access to a range of events relevant to human prenatal and neonatal brain development.

**Figure 2:**
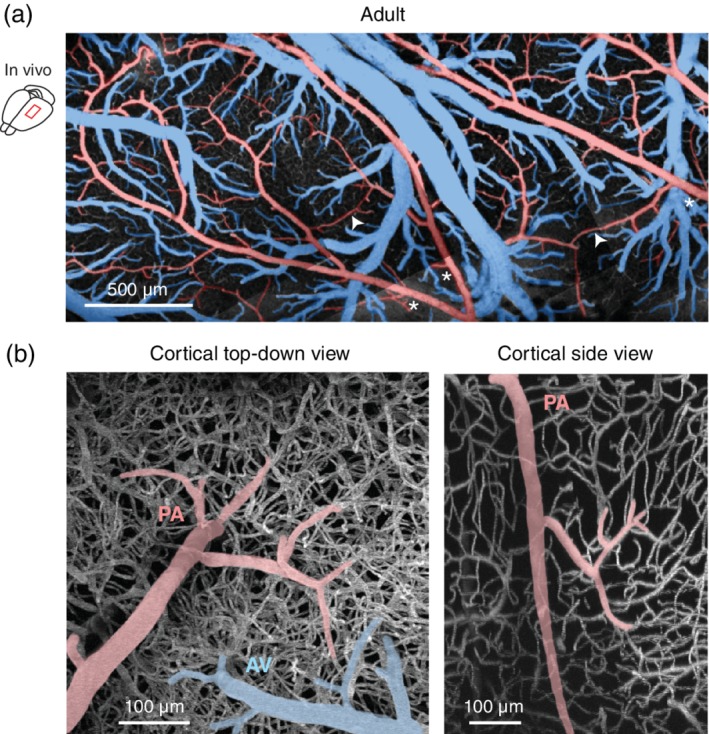
Microvasculature of the adult rodent cortex captured by in vivo imaging. (a) An image of the cerebral cortex surface captured by in vivo two‐photon microscopy through a cranial window in an adult rat. Pial arteriole networks are pseudocolored in red, and pial venular networks are in blue. Major branches of the middle cerebral artery traverse the entirety of the window (white asterisks), and smaller anastomotic connections link these major branches to create an interconnected web (white arrowheads; two examples shown). (b, left) Highly magnified view of a pial arteriole descending into the parenchyma as a penetrating arteriole (PA; red), and an ascending venule (AV; blue) emerging from cortex and draining into a pial venule in adult mouse cortex. The dense subsurface capillary network (white) is also visible in this image because the image is a projection across 700 μm of total cortical depth. (b, right) The same penetrating arteriole in the left panel is shown from a side view, revealing its descent into the cortex. A precapillary arteriole branches from the penetrating arteriole and ramifies into the capillary network

On a broader structural level, pial arterioles exist as an interconnected two‐dimensional network composed of the branches of the major cerebral arteries (middle, anterior, or posterior cerebral arteries), and shorter anastomotic connections linking these major arterial branches (Figure 2a, red; Blinder, Shih, Rafie, & Kleinfeld, 2010). Collateral arterioles further connect the distal ends of the cerebral arteriole territories to link the perfusion across distinct arterial domains. The pial arteriole network offers a number of intriguing attributes. First, it facilitates the distribution of blood supply across the cortex when serving the demands of local neuronal activity, i.e., neurovascular coupling. That is, blood flow can be more easily shifted from less active tissues to tissues with metabolic need through a multitude of flow routes (Devor et al., 2007). Second, in the case of brain ischemia due to focal arterial occlusion, the anastomotic connections allow blood to be rerouted from normally flowing arterioles into the under‐perfused tissues (Schaffer et al., 2006; Shih et al., 2009).

Penetrating arterioles branch from the leptomeningeal network and plunge into the cortical depth to bridge blood flow from the brain surface with capillary networks of the brain parenchyma (Figures 1b and 2b) (Nishimura, Schaffer, Friedman, Lyden, & Kleinfeld, 2007). Penetrating arterioles range in diameter from 10 to 30 μm, and like pial arterioles, are composed of an endothelial layer surrounded by a single cell thick layer of smooth muscle. They are surrounded by a cerebrospinal fluid‐filled Virchow‐Robin space in the upper layers of cortex, which is a continuation of the subarachnoid space (Jones, 1970; Roggendorf & Cervos‐Navarro, 1977). As the penetrating arteriole descends into cortex, the Virchow‐Robin space disappears and the glial limitans is formed as the end‐feet of parenchymal astrocytes come in close apposition to the vessel wall. Penetrating arterioles are also highly dynamic in their response to neuronal activity, but the conveyance of signals during neurovascular coupling differs from that of pial arterioles, and remains an active area of investigation (Iadecola, 2017).

Precapillary arterioles branch from the penetrating arterioles at different depths below the pial surface (Figures 1c and 2b). Precapillary arterioles are smaller in diameter (5–8 μm) than the parent penetrating arteriole. They are covered by contractile mural cells with a hybrid morphology of smooth muscle cells and pericytes (termed ensheathing pericytes in our past work) (Grant et al., 2017). This zone is relatively small and occupies between 1–4 initial branch orders before the microvasculature further ramifies into a dense 3D network of capillaries. Capillaries are the predominant vessel type in the brain, representing more than 90% of the total cerebrovascular length, and interweave with neurons and glia of the brain parenchyma to facilitate much of the nutrient and gas exchange (Figures 1d and 2b). They are composed of thin endothelial tubes (average 4 μm in diameter), covered by true capillary pericytes, with protruding ovoid cell bodies that extend long thin processes (Hartmann et al., 2015).

After transiting the capillary bed, blood is then routed back to the brain surface via postcapillary venules that empty into ascending venules, which have thin, stellate mural cells that incompletely cover the vascular wall (Figures 1e and 2b, left; Hartmann, Underly, Grant, et al., 2015). At the pial surface, the blood circulates through a network of venules that are interconnected much like the leptomeningeal arteriolar system, but still maintain a tree‐like structure (Figure 2a, blue). In the rodent brain, there are 2–3 ascending venules for every penetrating arteriole, suggesting multiple outputs for blood from the brain parenchyma (Nguyen, Nishimura, Iadecola, & Schaffer, 2007; Shih et al., 2013). Blood then empties into pial venules and larger draining veins and sinuses on the brain surface.

The cortical vasculature has been studied across multiple species, from rodent (Blinder et al., 2010; Blinder et al., 2013) and cats (O'Herron et al., 2016), to nonhuman primates (Weber, Keller, Reichold, & Logothetis, 2008) and human (Duvernoy, Delon, & Vannson, 1981; Lauwers, Cassot, Lauwers‐Cances, Puwanarajah, & Duvernoy, 2008). Remarkably, apart from some differences such as arteriole: venous ratio (Hartmann, Hyacinth, Liao, & Shih, 2017), the same hierarchical organization of vessels seems to be preserved across phylogeny. Thus, the basic building principles and mechanisms for blood allocation are similar between model organisms and the human brain.

## 3 DEVELOPMENT OF MICROVASCULAR ARCHITECTURE

How the vasculature is shaped during brain development remains less clear, although a good amount of knowledge has amassed (Lee, Han, Bai, & Kim, 2009; Tata, Ruhrberg, & Fantin, 2015). New vessels can be generated by two different processes, vasculogenesis and angiogenesis. Vasculogenesis is the process by which new vessels are formed de novo from vascular precursor cells (angioblasts). In contrast, angiogenesis corresponds to the growth and branching of existing blood vessels to expand the complexity of vascular networks. During embryogenesis, the process of vasculogenesis establishes the first vascular networks, which are then expanded via angiogenesis (Risser, Plouraboue, Cloetens, & Fonta, 2009).

In the rodent brain, vascularization starts with the formation of the perineural vascular plexus via vasculogenesis at the ventral neural tube at approximately embryonic day (E) 7.5–8.5 (Tata et al., 2015). Then, at E9.5, the first angiogenic vascular sprouts from perineural vascular plexus invade the neuroepithelium from the pial surface. The invasion is followed by branching, arborization, and migration of vascular sprouts from the pial network toward the ventricles where angiogenic factors, such as vascular endothelial growth factor (VEGF), are highly expressed (Bautch & James, 2009; Tata et al., 2015). Hypoxia is the principal regulator of VEGF expression, as it is a direct transcriptional target of both hypoxia‐inducible factors (HIF)‐1α and HIF‐2α (Forsythe et al., 1996; Levy, Levy, Wegner, & Goldberg, 1995). Deficiency in HIF‐1α and its dimerization partner HIF‐1β results in embryonic lethality at around E9.5 and E10.5, with severe defects in vessel formation, particularly in the yolk sac (Maltepe, Schmidt, Baunoch, Bradfield, & Simon, 1997; Ryan, Lo, & Johnson, 1998). During normal brain development, oxygenation is lower in the nonvascularized cortex at E10.5 than in the already vascularized ventral forebrain, and oxygen levels increase as the cortex becomes vascularized (Lange et al., 2016). However, there is a scarcity of data on how tissue oxygen content contributes to vascular growth postnatally.

At the time of birth, the pattern of interconnected pial arterioles has resemblance to what it will become in the adult brain (Figures 1c and 2; Bär, Miodoński, & Budi Santoso, 1986; Letourneur, Chen, Waterman, & Drew, 2014; Wang, Blocher, Spence, Rovainen, & Woolsey, 1992). Major branches of the cerebral arterioles are present, and the arterial tree has many short anastomotic connections, as seen in the adult brain. Further, collaterals arterioles that link the distal ends of separate cerebral arteriole domains are already present, as their formation occurs between E15.5 and E18.5 (Chalothorn & Faber, 2010). Postnatal remodeling of the pial vasculature primarily involves pruning about one‐half the anastomotic connections within the branches of the arterial trees, as well as pruning of collaterals linking the major cerebral arteries (Chalothorn & Faber, 2010). At P7, the pial arterioles alter their diameter, possibly due to adjustments in vascular tone, and lengthen to accommodate the growing cortex.

Interestingly, large differences in collateral artery density are seen between certain strains of mice, underscoring the importance of genetic factors that govern vascular patterning and density. In particular, Faber and colleagues have shown that BALB/c mice exhibit 60% fewer collaterals between middle and anterior cerebral artery territories than C57Bl/6 mice (Chalothorn & Faber, 2010). This difference was a result of reduced collateral formation during embryogenesis rather than postnatal vascular pruning. Subsequent studies have identified genes, such as VEGF‐A (Clayton, Chalothorn, & Faber, 2008) and chloride intracellular channel‐4 (Chalothorn, Zhang, Smith, Edwards, & Faber, 2015), that positively regulate collaterogenesis and increase collateral density during adulthood. Collaterals are critical for maintenance of blood supply in occlusive disease, and accordingly, mouse strains endowed with greater collateral density are more resilient to stroke injury caused by middle cerebral artery obstruction (Zhang, Prabhakar, Sealock, & Faber, 2010). Understanding the genetic basis of collaterogenesis may yield clinical biomarkers to identify patients with higher susceptibility to stroke injury due to collateral deficiency, and provide a means to harness collaterogenesis for stroke prevention and treatment.

**Figure 3:**
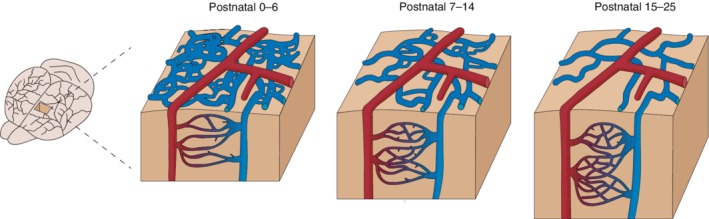
Postnatal remodeling of cortical vasculature in rodents: Refinement above, expansion below. Arteries are represented in red and veins in blue. At birth, the pial surface is covered by a dense plexus of veins that undergoes pruning over the initial few weeks postnatally. Pial arteriolar structure is generally unchanged, but there is regression of some anastomotic connections. Concurrently, the capillary bed undergoes massive proliferation through generation of new angiogenic sprouts. By postnatal Days 15–25, the capillary bed is almost formed and rates of endothelial proliferation decrease

**Figure 4:**
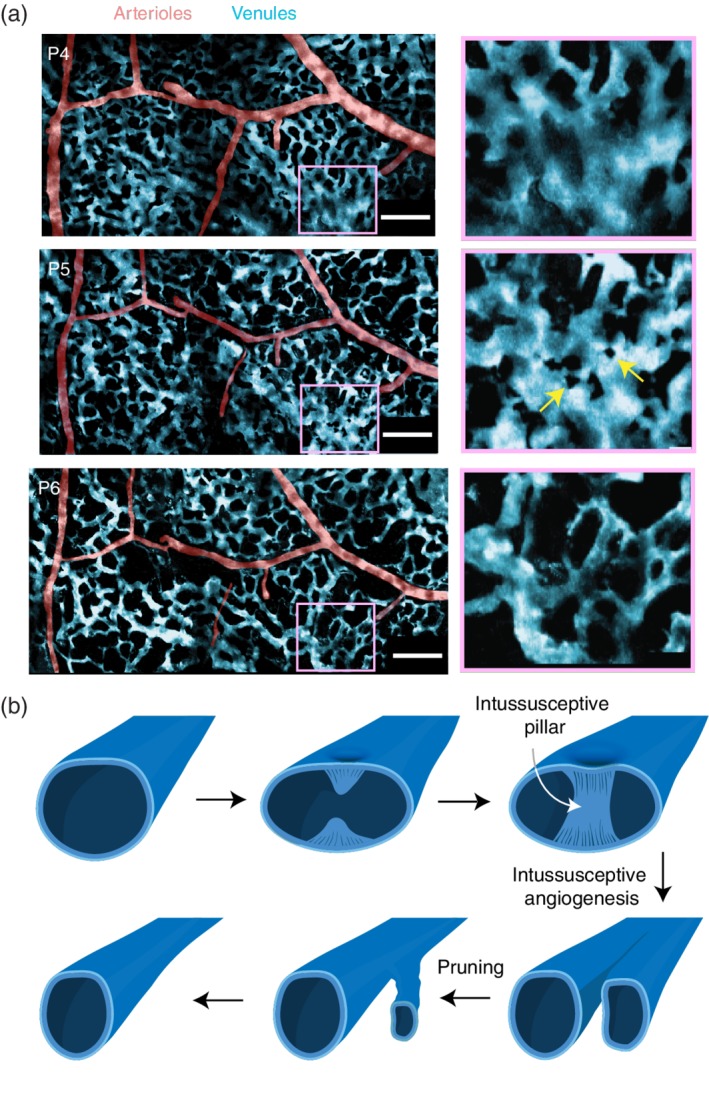
Evidence for intussusceptive remodeling in the developing pial vein plexus. (a) Image montages from in vivo two‐photon microscopy provide a wide‐field view of pial arteriole and venule remodeling between P4 and P6. Arteries are pseudocolored in red and veins in blue. The inset shows a region of the vein plexus that forms small holes (yellow arrows) that grow in size over a day. These regions are devoid of blood plasma because they do not label with an intravenously injected fluorescent dye that enables visualization during microscopy, consistent with an intussusceptive pillar. Images adapted from Letourneur et al. (2014). (b) A schematic depicting a hypothesized mechanism for venous remodeling in the developing mammalian cerebral cortex, involving intussusceptive angiogenesis and then pruning

In contrast to arterioles, the pial venular network begins as a dense vascular plexus covering most of the cortical surface at birth (Figures 3 and 4a; Fehér, Schulte, Weigle, Kampine, & Hudetz, 1996; Letourneur et al., 2014; Wang et al., 1992). This plexus is composed of small diameter, short distance loops that have been referred to as a superficial “capillary‐like” network, although they appear distinct from true parenchymal capillaries (Wang et al., 1992). By P7–P14, this dense plexus is then gradually pruned away in regions intervening the major draining venules. As a result, the branches of the venous network become more evident and blood flow within the remaining vessels increases to more effectively drain blood.

The remodeling of pial venules during brain development bears marked resemblance to vascular changes in the developing mammalian lung and the avian chorioallantoic membrane (CAM) (Ackermann et al., 2014; Burri, Hlushchuk, & Djonov, 2004). The key process to note is intussusceptive angiogenesis, where two indentations on opposing sides of the lumen grow and gradually connect to create a cylindrical pillar‐like structure within the lumen. This results in the creation of two daughter branches separated by the pillar. Intussusceptive pillars may then further widen to change the structure of the two daughter vessels. Imaging data from brain suggest the presence of similar events occur during pial venule development (Letourneur et al., 2014), evident as the formation of small black “holes” in a vascular tube (Figure 4a,b), similar to that noted by imaging studies of Djonov and colleagues in the chicken CAM (Djonov, Galli, & Burri, 2000), and more recently in the zebrafish caudal vein plexus (Karthik et al., 2018). However, rather than increase vascular network complexity, this intussusceptive process results in pruning of the initially amorphous venous plexus into a hierarchical tree‐like structure for effective blood drainage.

The transduction of mechanical forces by blood flow and pressure can strongly influence the remodeling process through alteration of endothelial structure and organization (Ranade et al., 2014; Skalak & Price, 1996). By collecting detailed vasodynamic information with repeated two‐photon imaging, Letourneur et al. found a positive relationship between the shear rate of blood flow, a metric proportional to shear stress force, and the subsequent diameter of venules 1 day later. That is, venules with low shear rate were likely to be pruned away, while those with high shear rate widened and stabilized within the network (Letourneur et al., 2014). Similarly, repeated imaging of mid‐brain microvessels in zebrafish revealed that flow in vessels destined to be pruned was lower and more variable that in microvessels that persisted within the network (Q. Chen et al., 2012). Flow‐dependent pruning must be essential for removal of redundant connections and promotion of efficient routing of cerebral blood flow. Since, improper venous remodeling can impact cerebral circulation by impairing cerebrovascular output, further studies on how mechanical forces govern cerebrovascular development are warranted.

Beneath the brain surface, the penetrating arterioles and ascending venules are present at birth, although their characteristics continue to be refined. The penetrating arterioles are initially thin and capillary‐like in diameter within the first few days after birth, but then their lumen expand and walls muscularize to become more arteriole‐like by P10 (Rowan & Maxwell, 1981). Penetrating arterioles give off branches to perfuse downstream capillaries. However, there is little data on the development of the precapillary arteriole zone, which recent studies have shown to be critical for the initiation of neurovascular coupling (Hall et al., 2014; Rungta, Chaigneau, Osmanski, & Charpak, 2018).

The capillary network at birth is sparse and incomplete compared to the dense networks of the adult brain (Figure 3). The numerous ongoing angiogenic and regression events result in non‐flowing endothelial tubes that may contain only a trickle of blood plasma until they become patent. Over the course of weeks, the cortical capillary network undergoes dramatic expansion via angiogenesis (Harb et al., 2012; Norman & O'Kusky, 1986; Risser et al., 2009). The length of capillary branches and individual endothelial cells also elongate to keep pace with the rapidly expanding cortical volume (Keep & Jones, 1990). Detailed histological analyses of endothelial sprouting have revealed bursts of angiogenesis that occur at different stages and cortical layers (Rowan & Maxwell, 1981). From P5 to P25, new capillary loops are often formed by short distance sprouting without the need for proliferating endothelial cells. That is, single endothelial tip cells can reach to connect with other capillary branches through the extension of their cellular processes (Harb et al., 2012). In vivo imaging of postnatal vascular development has revealed that many of the nascent angiogenic sprouts are eliminated, with only a small subset forming lasting connections within the capillary network (Harb et al., 2012). However, it was not clear from data of Harb et al. whether eliminated vessels were true capillaries, or “capillary‐like” venules near the brain surface, which are known to regress as discussed above. Nevertheless, capillary refinement by regression must still be greatly outweighed by angiogenesis. By P15–P25, the extent of capillary angiogenesis begins to subside and capillary density stabilizes (Harb et al., 2012; Walchli et al., 2015; Zeller, Vogel, & Kuschinsky, 1996). During this stabilization, the proliferation rates of both pericytes and endothelial cells decline (Harb et al., 2012).

In light of data from other vertebrate models (zebrafish; Bussmann, Wolfe, & Siekmann, 2011) and mammalian organs (heart and retina; Red‐Horse, Ueno, Weissman, & Krasnow, 2010; C. Xu et al., 2014) showing that angiogenic sprouts tend to emerge from venules, the microvascular zone in which nascent angiogenic sprouts emerge in the postnatal brain needs further characterization. Venous sprouts can in fact give rise to arterioles through migration and reprogramming of endothelial cells, although in the mammalian postnatal brain this seems to be less likely with arterioles pial and penetrating arteriole structures already defined. The underlying reason for why veins are a source of angiogenic sprouts remains obscure. Decreased tendency to rupture due to lower intravascular pressure (Red‐Horse & Siekmann, 2019), or decreased oxygen content surrounding venules are logical hypotheses for this phenomenon.

Gray and white matter tissues require different levels of metabolic supply. It is estimated that white matter, which is densely packed with energy efficient myelinated axons, requires 1/4–1/3 the energy used by gray matter. It is therefore not surprising that capillary networks of white matter are less dense compared to gray matter. Interestingly, developmental studies have revealed that capillary density in gray matter doubles during the period of birth to P20, while capillary density in white matter structures increases to a far lesser extent, or even decrease with age (Zeller et al., 1996).

## 4 POSTNATAL REFINEMENT OF THE BBB

**Figure 5:**
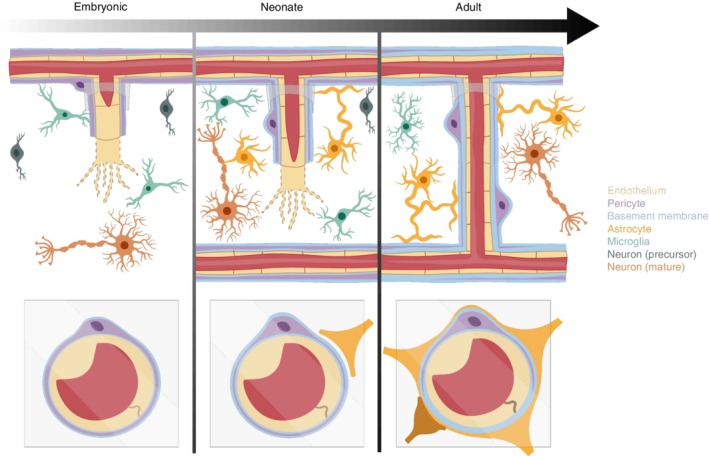
Maturation of the neurogliovascular unit. The upper panels are a schematic depicting the gradual integration of different cell types and the basement membrane in brain capillaries. The lower panels show a cross‐sectional view of the capillary wall at each developmental stage. Developmental periods (embryonic and neonate) show an angiogenic sprout with extensive filopodia. This sprout connects with an existing part of the capillary network and becomes patent. Increased tight junction density, basement membrane thickness, and astrocytic end‐foot investment contributes to BBB maturation

The capillary endothelium creates an efficient physical and biochemical barrier between the blood and the brain (Cardoso, Brites, & Brito, 2010). Through tight regulation of intracellular trafficking and dynamics of intercellular junctions, endothelial cells control several aspects of BBB function, including transport of micronutrients and macronutrients, receptor‐mediated signaling, leukocyte trafficking, and osmoregulation. The multistep process of BBB development does not occur with endothelial cells alone (Figure 5). The recruitment and cross talk of other cell types (pericytes, microglia, astrocytes, and neurons) in the neurogliovascular unit are required for refinement and maturation of the BBB. In particular, astrocytes are only fully integrated after birth suggesting that BBB development extends into the postnatal period in rodents. Excellent reviews have been written about the complex molecular and cellular events involved in barriergenesis (Lee et al., 2009; Obermeier, Daneman, & Ransohoff, 2013; Saunders, Liddelow, & Dziegielewska, 2012), much of which has been characterized during embryogenesis. Here, we aim to highlight some aspects of this process that suggests that BBB maturation continues to occur postnatally.

When endothelial cells first invade the CNS, there are features of endothelial permeability, including fenestrae, upregulated rates of transcytosis (J. Xu & Ling, 1994; Yoshida, Yamada, Wakabayashi, & Ikuta, 1988), and also high levels of leukocyte adhesion molecules (Daneman, Zhou, Kebede, & Barres, 2010). While endothelial tight junction structures are already present at E12, detailed ultrastructural analyses in late embryonic (E15–E18) to postnatal stages (P1) have revealed an increase in their density and complexity until adulthood (Daneman et al., 2010; Kniesel, Risau, & Wolburg, 1996). Measurements of transendothelial electrical resistance and vascular permeability in situ from fetal to postnatal rat brain vasculature revealed a gradual sealing of the BBB from E17 to P33 (Butt, Jones, & Abbott, 1990). Ultrastructural analyses of capillary walls in rat corpus callosum from P1 through P7, which were already covered by pericytes, showed a numerous pinocytotic vesicles with high permeability to intravenously injected ferritin. At approximately P14, there is a decrease in vesicle abundance and ferritin leakage, concomitant with increasing coverage of the capillary wall by astrocytic endfeet. During this maturation, the pericapillary spaces are reduced in size and replaced by an astrocytic–basement membrane interface (J. Xu & Ling, 1994).

Endothelial expression of the efflux transporter Pg‐P (P8) (Daneman et al., 2010) and ABC‐transporters (Ek et al., 2010) increases during postnatal development, indicating greater capacity for molecular efflux from the brain. The glucose transporter, GLUT1, is already expressed in early embryonic stages in capillary tight barrier structures. However, major changes in the regional pattern of GLUT1 distribution occur at P3 until 2‐months of age (Harik et al., 1993; Zeller et al., 1996). Moreover, combined FACS purification with GeneChip analysis was used to generate a transcriptional profile of highly purified CNS endothelial cells across different ages. Comparing the ages ranges of P2–P8 with P60–P70, endothelial cells exhibited differences in genes that control angiogenesis and formation of the BBB, suggesting a continued maturation of the BBB postnatally (Daneman et al., 2010).

## 5 BUILDING THE WALL: FROM A VASCULAR NETWORK TO A NEUROGLIOVASCULAR UNIT

The assembly of the neurogliovascular unit involves interaction with pericytes, astrocytes, neurons, and noncellular structural components such as the basement membrane (Figure 5). This process initiates at embryonic stages, but is completed in early postnatal stages of development. Below, we discuss the multicellular dynamics that occur postnatally to form a functional vascular network.

### 5.1 Pericyte‐endothelial dynamics: Angiogenesis and vessel stabilization

Pericytes are mural cells embedded in the basement membrane of brain capillaries, directly abluminal to the endothelium (Sweeney, Ayyadurai, & Zlokovic, 2016). Communication between the endothelial cells and pericytes is essential for the establishment of a number of cerebrovascular functions, including vascular structure and BBB integrity. This communication is mediated by signaling at direct cell–cell contacts occurring within peg and socket interactions, as well as through the release of growth factors and the modulation of the extracellular matrix. Pericytes may also be linked to the endothelium through gap junctions, tight junctions, and focal adhesions junctions (Armulik, Abramsson, & Betsholtz, 2005). One ultrastructural imaging study revealed pericyte–endothelial “gap junctions” in embryonic rat brain capillaries (Fujimoto, 1995), which may be required for endothelium‐induced differentiation of mural cells (Hirschi, Burt, Hirschi, & Dai, 2003). Numerous signaling pathways underlie pericyte–endothelial cross talk for the development of vascular structure and barriergenesis, and these have been reviewed in detail previously (Armulik et al., 2005).

Pericytes are present on the brain endothelium as early as E10, and contribute to a large expansion of capillaries that occurs between E14.5 and E18.5 (Jung, Arnold, Raschperger, Gaengel, & Betsholtz, 2017). These early pericytes or pericyte progenitors express established pericyte marker genes *Pdgfrb* and *Cspg4*, but still lack other markers such as *Anpep* (CD13) seen in the adult brain, suggesting an ongoing maturation of their phenotype. At the time of birth, the morphological characteristics of brain pericytes are distinct from that in the adult brain (Figure 5). The stereotyped protruding cell bodies of pericytes are less conspicuous, and their cellular processes fully enwrap the endothelium, as opposed to the partial coverage seen in adulthood (J. Xu & Ling, 1994). It is widely appreciated that pericytes promote quiescence and inhibition of endothelial growth (Durham, Surks, Dulmovits, & Herman, 2014; Orlidge & D'Amore, 1987). However, in early development, pericytes must also support rapid capillary network expansion. Indeed, a recent study showed that pericyte ablation in postnatal retina led to impaired endothelial sprouting and patterning via disruption of local VEGF/VEGFR signaling between pericytes and endothelial cells (Eilken et al., 2017).

There are two seemingly opposing views to how pericytes and endothelial cells coordinate the formation of new capillaries during sprouting angiogenesis. The predominant concept is that endothelial cells lead the process, and pericytes lag behind. Endothelial tip cells first migrate from existing capillary networks through a VEGF‐dependent mechanism, and penetrate into the tissue (Gerhardt et al., 2003). Endothelial stalk cells then follow the tip cell and proliferate to further extend the angiogenic sprout. Tip cells release the growth factor PDGF‐B, which is deposited in dimeric form (PDGF‐BB) on the surrounding vascular extracellular matrix and forms the signal for pericyte recruitment to the nascent endothelial tube, leading to subsequent vessel stabilization and maturation (Hellström et al., 2001; Lindahl, Johansson, Leve, & Betsholtz, 1997; Lindblom et al., 2003). Cross talk between pericytes and endothelial cells then promotes vascular stability and increase in BBB integrity by increasing expression of endothelial tight junction proteins and reducing transcellular transport through caveolae (Armulik et al., 2010; Daneman et al., 2010). This concept suggests that there is a window of endothelial plasticity and BBB immaturity until pericytes are invested into new capillaries. In retina, this window can be significant, lasting up to several days, before pericytes invade the endothelial plexus (Benjamin, Hemo, & Keshet, 1998).

A second view is that pericytes lead the angiogenic process by invading the parenchyma, followed in turn by endothelial cells (Amselgruber, Schafer, & Sinowatz, 1999). In this case, pericytes may be a source of VEGF that provides the guiding signal for endothelial cells (Reynolds, Grazul‐Bilska, & Redmer, 2000). This concept implies that pericytes are already present on angiogenic sprouts and capable of delivering signals to rapidly promote BBB integrity and vessel stabilization, thereby limiting the window for vessel immaturity. It further raises the possibility that pericytes have some role in deciding the location of new angiogenic sprouts in existing capillary networks, and in guiding or bridging the sprout through the brain parenchyma. This mechanism may be more relevant to angiogenesis occurring in vascular networks already abundant in pericytes. However, these opposing views come from diverse model systems and organs, and likely differ in various angiogenic contexts. Recent data from Payne et al. reveal a potential middle ground where pericyte and endothelial tip cells co‐migrate, taking turns to lead the new capillary sprout (Payne et al., 2019). The relative position of pericytes and endothelial cells was hypothesized to be dependent upon fluctuations in the availability of ligands such as PDGF‐BB at the angiogenic front.

While most of the vascular growth within the capillary network is attributed to sprouting angiogenesis, a second means to increase capillary network complexity is through intussusception or “splitting angiogenesis” (Burri et al., 2004). While, intussusceptive angiogenesis has been characterized in other developing organs (lung) and may occur in pial venules, it remains unclear whether it contributes significantly to development of brain capillary networks.

Pericytes contact the majority of the endothelium via long processes that run longitudinally along the vessel axis. These processes allow a single pericyte to contact and communicate with hundreds of micrometers of capillary length (Hartmann, Underly, Grant, et al., 2015). The processes incompletely wrap the underlying endothelium, and studies often report the extent of this overlap as “coverage,” which ranges ~50–70% coverage in the normal brain. The extent to which pericytes cover the endothelium is developmentally regulated by PDGF‐B/PDGFRbeta signaling (Hellström, Kalén, Lindahl, Abramsson, & Betsholtz, 1999). While mice that are null for the *Pdgfb* or *Pdgfrb* genes have near complete absence of brain pericytes, and are perinatal lethal (Lindahl et al., 1997; Soriano, 1994), mice experiencing partial disruption in PDGF‐B/PDGFRbeta signaling are viable and useful for postnatal developmental studies. For example, PDGFB^ret/ret^ mice harbor a mutation to PDGF‐B that inhibits ligand binding to the vascular basement membrane, thereby removing the signal for pericyte recruitment. These mice have pericyte coverage of only 25% of the endothelium and exhibit aberrant capillary dilations and increased transcellular BBB leakage (Armulik et al., 2010). They also exhibit fewer branch points in the capillary network, consistent with a role for pericytes in capillary patterning. Using hypomorphs that collectively exhibit a range of severities in pericyte loss, pericyte coverage was found to correlate with BBB integrity, where greater pericyte loss was associated with worsened leakage through transcellular routes (Daneman et al., 2010). Transcriptomic studies further revealed that pericytes suppressed pathways for endothelial leakage, including genes involved in promotion of immune cell trafficking.

Pericytes also regulate endothelial expression of major facilitator super family domain containing 2a (MFSD2A), and lipid transporting protein at the plasma membrane. Genetic ablation of *Mfsd2a* leads to BBB leakage due to increased transcytosis from embryonic to adult stages (Ben‐Zvi et al., 2014). Recent work also showed that the spatiotemporal expression of a cell adhesion receptor, CD146, is important for pericyte recruitment/attachment and BBB maturation (J. Chen et al., 2017). Pericyte‐specific deletion of CD146 causes impairment in pericyte coverage and BBB breakdown.

### 5.2 The vascular basement membrane: More than just cement

The microvascular basement membrane is a thin layer of extracellular matrix, composed of structural proteins, glycoproteins, and proteoglycans, located between the endothelium and perivascular end‐feet of the parenchyma. Pericytes that are located within this intermediate space are completely embedded within the basement membrane. The basement membrane anchors cells within the neurogliovascular unit and forms a physical barrier to regulate cellular migration, such as leukocyte trafficking. The basement membrane also facilitates cell–cell communication at the vascular wall. For example, it provides a scaffold to retain signaling molecules such as PDGF‐BB and VEGF crucial for angiogenesis and pericyte recruitment. Bioactive components such as laminin are also required for cell differentiation and migration. The basement membrane is evident as early as E20 in rat embryos but still ill‐defined at birth. Its density and thickness increases with postnatal development suggesting continued formation by perivascular cells (Figure 5; Donahue & Pappas, 1961; J. Xu & Ling, 1994). In mice, laminin and agrin are the earliest proteins to appear at the vascular wall around P0, reaching the peak levels as early as P7 (Lunde et al., 2015). This early basement membrane and its junctions with astrocytic endfeet become critical for establishing the polarization of proteins, such as the water channel aquaporin‐4 (AQP4), at the vascular interface.

Highlighting the importance of basement membrane for vascular integrity, alterations, and disruption of the basement membrane can lead to changes in the endothelial cell cytoskeleton, which in turn affects tight junction structure and BBB integrity. Accordingly, laminin α2 subunit‐null mice at P21 exhibit BBB hyperpermeability correlated with decreases in VE‐cadherin, claudin‐5, and occludin, as well as lower pericyte and astrocytic end‐foot coverage (Menezes et al., 2014). Yao and colleagues showed that the laminin produced specifically by astrocytes is needed for polarization of astrocytic end feet, pericyte differentiation, and maintenance of BBB properties (Yao, Chen, Norris, & Strickland, 2014). Additionally, it has been shown that endothelial laminin, laminin α4, regulates vascular integrity at embryonic and neonatal stages, and its deletion leads to impaired microvessel maturation causing hemorrhages (Thyboll et al., 2002). Mutations in collagen type IV, the major component of the basement membrane, similarly leads intracerebral hemorrhage in both mice and humans during development (Gould et al., 2005).

### 5.3 Astrocyte‐endothelial cross talk: Barriergenesis and vascular remodeling

In the adult brain, astrocytes form a layer of fine lamellae, called end‐feet, that is closely opposed to the abluminal surface of the endothelium and basement membrane (Abbott, Ronnback, & Hansson, 2006). This layer forms a physical barrier at the vascular wall, and also facilitates the neurovascular cross talk needed for BBB maturation. Astrocytes first become prominent during the perinatal period, coincident with the onset of expansion of cortical capillary networks (Figure 5; Ma, Kwon, & Huang, 2012; Marshall, Suzuki, & Goldman, 2003). Some findings show that after populating the cortex, astrocytes continue to proliferate locally during the first 3‐weeks of postnatal development (Ge, Miyawaki, Gage, Jan, & Jan, 2012). In the rat brain, increase in astrocyte density proceeds until P50, when adult density levels are reached (Seregi, Keller, & Hertting, 1987; Stichel, Muller, & Zilles, 1991). Also, end‐foot expression of AQP4, a channel for water transport across the BBB with implications in glymphatic drainage, is weakly expressed at P7 and shows a sharp increase in expression toward adulthood (Agre et al., 2002; Iliff et al., 2012; Lunde et al., 2015). It has been suggested that pericytes are needed to attract or maintain astrocyte end‐foot coverage of the vessels, as pericyte‐deficient mice exhibit defects in association and polarization of astrocytic end‐feet (Armulik et al., 2010).

Astrocytes seem to also serve a critical role in regulating vascular architecture as they interact closely with blood vessels by P5–P7, and the inhibition of their proliferation results in a drastic reduction in the density and branching of cortical blood vessels (Ma et al., 2012). Loss of astrocyte investment also leads to abnormal endothelial proliferation and enlargement of vessel diameter. Further, the expression level of several of the proteins normally present at the perivascular astrocyte end‐feet is very low in immature astrocytes, including the canonical astrocyte marker glial fibrillary acidic protein (Lunde et al., 2015).

Astrocytes also orchestrate the postnatal formation and reorganization of vascular scaffolds for cellular migration. Detailed gain and loss of function studies have shown that astrocyte release of VEGF influences the formation of blood vessels in the rostral migratory stream, which is used as a scaffold for neuroblasts migrating to the olfactory bulb (Bozoyan, Khlghatyan, & Saghatelyan, 2012). In vivo downregulation of VEGF specifically in the astrocytes affects vascular development and leads to complications in neuronal migration. Curiously, postnatal expression of VEGF by astrocytes is unique to the rostral migratory stream, as expression in cerebral cortex is higher in neurons at early stages of development (Bozoyan et al., 2012). However, as vascular beds stabilize around P24, the proportion of astrocytes expressing VEGF increases in cortex relative to neurons (Ogunshola et al., 2000).

### 5.4 Microglia‐endothelial contact

Microglia are the resident innate immune cells of the brain, and represent ~10–15% of all glia in the brain (Nayak, Roth, & McGavern, 2014). Studies from developing brain and retina suggest that microglia cells colonize prior to tissue vascularization, but then come in close contact with the microvasculature during the vasculogenic and angiogenic process (Tammela et al., 2011). Microglia interact with endothelial tip cells to coordinate anastomotic connections within the capillary bed (Figure 4). They are often seen at sites where two tip cells with filopodia come in close contact and anastomotic connections are likely to occur (Fantin et al., 2010). Rymo and colleagues further probed the role of microglia in combined in vivo and ex vivo studies of mouse retina and an aortic ring model (Rymo et al., 2011). First, by examining mice with microglial deficiency (M‐CSF/CSF‐1 deficient mice), they showed that microglial absence led to a sparser vascular network. Then using aortic ring cultures, they showed that microglia could stimulate vessel sprouting and branching via release of a soluble, microglial‐derived molecule. Together, this indicates a bidirectional communication between microglia and endothelial tip cells during the formation of new vascular connections. With respect to underlying mechanisms of microglia–endothelial communication, work from Tammela and colleagues suggested that a subpopulation of microglial cells express VEGF‐C, which is able to activate VEGFR‐3 in tip cells to reinforce Notch signaling (Tammela et al., 2011). This contributes to a phenotype change of the endothelial cell at sites of vascular fusion.

In addition to roles in regulation of vascular growth, microglial cells associated with brain capillaries in the early postnatal stage exhibit a phagocytic, amoeboid morphology. Over a period of 3‐weeks, this shape then shifts to the classic morphological profile of ramified, resting surveillant microglial seen in the normal adult brain (Arcuri, Mecca, Bianchi, Giambanco, & Donato, 2017; Zusso et al., 2012). Amoeboid microglia are able to uptake and retain intravenously injected dyes, suggesting that they serve as frontline protection from exogenous substances that may enter the pericapillary space by transendothelial transport during brain development (J. Xu & Ling, 1994).

### 5.5 Neuronal activity alters the development of capillary structure

The microvascular architecture of the brain is shaped by neural activity during the postnatal period (Lacoste et al., 2014; Whiteus, Freitas, & Grutzendler, 2014). Lacoste and colleagues showed that enhancement of neural activity between P0 and P5 by gentle whisker stimulation (15 min per day over 8 days) leads to increased vascular density and branching in the barrel field of sensory cortex (Lacoste et al., 2014). Conversely, vascular density and branching were decreased when vibrissal sensory input was reduced by genetic impairment of neurotransmitter release at thalamocortical synapses, or by whisker plucking. Whiteus and colleagues also examined the relation between neural activity and vascular structure and reported that excessive stimulation and repetitive neural activation during P15–P25 caused decreases in vascular density by reducing endothelial proliferation and sprouting (Whiteus et al., 2014). This effect was observed using a variety of stimulation paradigms, ranging from treadmill exercise to auditory stimulation. While these two seminal studies seem contradictory at first, the contrasting outcomes may be explained by the extent of stimulation given, where Lacoste et al. used more physiologically relevant stimulation and Whiteus et al. aimed to test the effects of overstimulation. It seems that physiological stimulation strengthens the neurovascular link, while overstimulation disrupts this link and leads to lasting deficits in microvascular density. In contrast to the plasticity of the capillary bed, a recent study demonstrated that the structure of pial arteriole networks was unaffected by sensory deprivation (Adams, Winder, Blinder, & Drew, 2018). The absence of vibrissal input from P2 to P30, due to continual whisker plucking, had no effect on the branching or anastomotic connections of pial arterioles or the density of penetrating arterioles, suggesting that this system is already set prior to birth.

The construction of vascular and neuronal networks is guided by similar mechanisms during development (Carmeliet & Tessier‐Lavigne, 2005). Many signaling molecules important for axonal guidance, such as netrins, semaphorins, slits, nogo, and ephrins, are also able to influence vascular growth through attractive or repulsive cues. For example, recent studies discovered that a membrane protein RTN4 (previously NOGO‐A), which inhibits axonal growth in adults, also acts as a negative regulator of angiogenesis during postnatal CNS development (Wälchli et al., 2013). RTN4 is expressed in neurons in the postnatal brain (P10), with close proximity to vasculature endothelial tip cells and their filopodial protrusions. Genetic ablation or antibody‐mediated neutralization of RTN4 in P4 or P8 mice led to a significant increase in the number of endothelial tip cells at P10, and the addition of new patent capillary branches in the microvascular network (Walchli et al., 2017). Another common pathway that influences both neural and vascular development is WNT signaling. WNTs are secreted glycoproteins with well‐established roles in development of neuronal circuitry (Inestrosa & Varela‐Nallar, 2015). In vascular development, WNT/β‐catenin signaling has dual effects on angiogenesis and BBB development through embryogenesis to postnatal periods. Loss of WNT 7a/b or WNT receptor, Frizzled 8, reduces vessel density and capillary bed structure, and also results in vascular leakage due to decreased tight junction protein expression and structural integrity (Daneman et al., 2009; Liebner et al., 2008; Zhou et al., 2014).

A well‐studied positive regulator of angiogenesis is VEGF signaling. As discussed above, in postnatal rats (P8–P13), VEGF is primarily expressed by neurons. Since VEGF release can be modulated by neural activity, it is can serve as a neurovascular link for regulation of angiogenesis (Kim et al., 2008). Between P13 and P24, VEGF expression decreases in neurons and increase in astrocytes. Since astrocytes are ideally positioned to sense neuronal activity and interact with the vasculature, this shift in expression may continue to shape vascular architecture concurrent with the maturation of astrocyte–endothelial signals required for vascular tone and neurovascular coupling (Tran, Peringod, & Gordon, 2018).

Functional imaging studies of the developing human brain have observed patterns of hemodynamic responses that differ from adult responses. In adults, the increase of local neural activity is nearly always accompanied by increases in local blood flow to the active brain area, that is, functional hyperemia. In contrast, the developing brain exhibits absent or even inverted vascular responses to neural activity (Kozberg, Ma, Shaik, Kim, & Hillman, 2016; Zehendner, Tsohataridis, Luhmann, & Yang, 2013). To better understand this phenomenon, Kozberg and colleagues examined the spatiotemporal dynamics of oxidative phosphorylation (inferred by fluorescence changes in flavoprotein) concurrent with neuronal activity in P7 mice (Kozberg et al., 2016). They concluded that neural activity led to local oxygen consumption, but concurrent vascular responses were insufficient to supply additional oxygen. This was postulated to create conditions of local tissue oxygen depletion, which could reduce thresholds for hypoxic injury, indicating a period of vulnerability during early development. On the other hand, local oxygen reduction may be necessary as an angiogenic trigger to link the most active brain regions with the greatest capillary density. Nevertheless, absolute levels of tissue oxygen reduction during brain activity in neonates remain to be measured in detail, and this could be facilitated with the advent of new phosphorescent tissue oxygen probes (Esipova et al., 2019).

New techniques in imaging of live mouse pups have created fertile ground for understanding how neurovascular coupling is established, concurrent with maturation of the vascular architecture and neurovascular unit. Indeed, capillary networks in the adult brain provide retrograde hyperpolarization for upstream arteriolar dilation, and the architecture needed to convey these endothelial signals may be lacking in early development (Longden et al., 2017). Further, the essential cellular elements for coupling, including astrocytes and pericytes, are also not fully integrated (Harris, Reynell, & Attwell, 2011).

## 6 OPEN QUESTIONS

Collective research on cerebrovascular development has emphasized key topics for future study. These include, but are not limited to:

1. *The mechanisms that establish the microarchitecture of brain capillaries*. Research suggests that developing capillary networks are modifiable by neural activation/inhibition in the perinatal period and that these signals set the branching pattern and density of capillaries that ultimately supply blood flow in the adult brain. It will be critical to understand the mechanism and endothelial cues underlying this approach for modulating capillary structure and function, and whether it can be harnessed to promote microcirculatory function in human disease.
2. *The basis of neurovascular coupling in the developing brain remains understudied*. Given that functional hyperemia is distinctly regulated in the infant brain, the interpretation of fMRI signals that rely on blood flow and oxygenation state as a surrogate for neural activity requires careful evaluation. Further, it is important to understand if the apparent lack of vascular reactivity despite robust neural activation creates conditions of oxygen deficiency in the infant brain. Does lowered oxygen content lead to increased vulnerability to hypoxia, or is this a normal angiogenic signal to link neuronal demand to vascular supply?
3. *The perinatal refinement of BBB integrity is poorly characterized*. It remains controversial as to whether humans are born with a fully functional BBB (Saunders et al., 2014). This is important to clarify because differences in permeability to drugs and other pharmacological agents may have inadvertent effects in treatments of nursing mothers and neonates. Further, while much is known about structural elements and genetics of the BBB, there remains limited information on the cross talk that occurs between cells of the neurovascular unit that leads to BBB maturation. In the developing brain, there are both signals that promote cell proliferation, as well as those that support vascular maturation. It remains unclear how these processes coexist to produce a functional vascular system.

## 7 A WINDOW TO VISUALIZE THE DEVELOPING BRAIN

**Figure 6:**
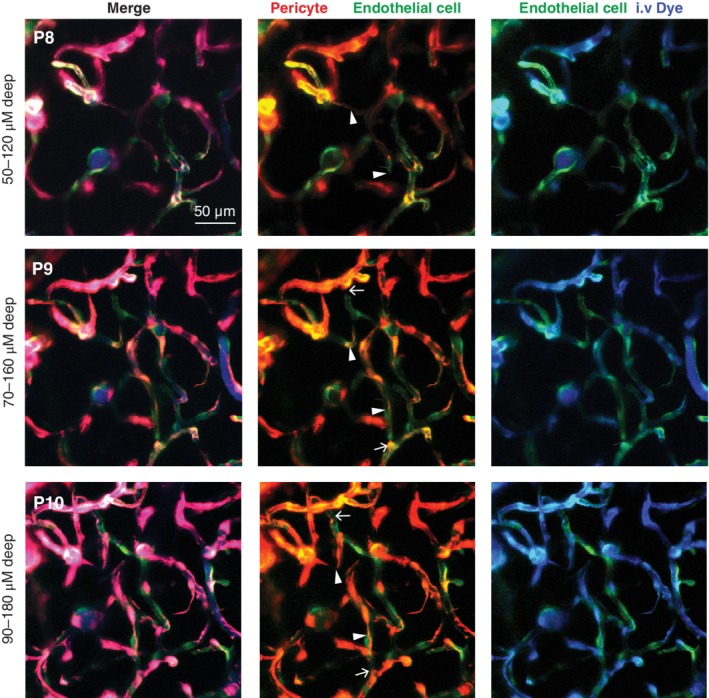
In vivo two‐photon imaging of capillary network expansion in the postnatal brain. (Upper row) Capillaries visualized through a chronic, thinned‐skull window in a P8 mouse pup. The mouse is double transgenic for Tie2‐GFP and PDGFRbeta‐tdTomato, allowing concurrently visualization of endothelial cells (green) and pericytes (red). An fluorescent dye (Alexa 680‐dextran; 2 MDa) was injected intravenously to label the blood plasma (i.v. dye). To aid in visualization, the middle panel shows only endothelial cells with pericytes, and the right panel shows only endothelial cells and the i.v. dye. Note that many capillaries are covered by pericytes, but some regions still lack pericyte coverage/contact (middle panel). Also, many capillaries have not yet lumenized, i.e., have not filled with the i.v. dye (right panel). Further, angiogenic sprouts are clearly visible in this expanding network (white arrowheads). (Middle row) The same region of cortex was reexamined 1 day later. The angiogenic sprouts have grown further and are now integrated into an existing part of the capillary network (white arrow). (Lower row) The nascent capillary branches formed over the previous 2 days are still present, and further increase in pericyte coverage (middle panel)

While mouse brain development has been extensively studied using histology and in vitro approaches, quantitative characterization of morphological changes over time remains a challenge. Ex vivo and in vitro studies are unable to replicate the complexity of neurovascular connections and the dynamics of perfused blood vessels in the intact brain. In vivo imaging through cranial imaging windows using two‐photon microscopy (TPM) has emerged in recent years as one approach to overcome this limitation (Harb et al., 2012; Letourneur et al., 2014). In addition to providing cellular to subcellular resolution of microvessels below the brain surface, TPM allows longitudinal assessment of physiological dynamics and remodeling over days (Figure 6). The use of thinned‐skull windows reduces the likelihood of inducing inflammation or exposing the brain to air, which can change the trajectory of cerebrovascular development (Letourneur et al., 2014). In combination with powerful genetic approaches currently available to fluorescently label and manipulate neurovascular cells (Hartmann, Underly, Watson, & Shih, 2015), TPM holds promise for understanding the orchestration of cerebrovascular development in vivo. It further allows the opportunity to track maturation of tight junction complexes, concurrent with assessments of BBB permeability (Knowland et al., 2014), and vascular reactivity to neuronal metabolic demand.

In vivo imaging provides an additional advantage of noninvasive optical manipulation of cell types at specific times in the developmental process to probe the effects of their absence during cerebrovascular development. Recent studies have shown the feasibility of precision optical ablation of a variety of cell types, including pericytes (Berthiaume et al., 2018; Hill, Damisah, Chen, Kwan, & Grutzendler, 2017), microglial cells (Lou et al., 2016), astrocytic end‐feet (Kubotera et al., 2019), and neurons (Hill et al., 2017) in the adult brain. This approach has yet to be applied to study the role of specific cell types during cerebrovascular development. It is a powerful alternative to genetic ablation studies, such as diphtheria receptor and toxin induced apoptosis, as it does not disrupt cell types in a global fashion, and does not rely on the availability of Cre drivers, which are often not entirely specific for the cell type of interest.

## 8 CONCLUSION

Research in cerebrovascular development has revealed that vascular architecture is remarkably plastic and modifiable in the first few weeks of like. This process involves refinement of vascular networks at the pial surface, concurrent with increasing growth and density of subsurface capillary networks. It has also demonstrated the complex interplay of cell types required to assemble a functional neurovascular unit. In collating the data, we have highlighted open questions worth examining with respect to development of neurovascular structure, reactivity, and integrity. Innovative in vivo imaging approaches have enabled researchers to begin tracking complex developmental processes in the living brain. A better understanding of cerebrovascular development will improve our knowledge of how the vasculature serves the immense task of feeding the brain in adulthood, and may also unveil approaches to leverage developmental programs for the improvement of vascular function in diseases of the adult and aging brain.

## CONFLICT OF INTEREST

The authors have declared no conflict of interest for this article.

## AUTHOR CONTRIBUTIONS

**Vanessa Coelho‐Santos**: Conceptualization‐equal; writing‐original draft‐equal; and writing‐review and editing‐equal. **Andy Shih**: Conceptualization‐equal; writing‐original draft‐equal; and writing‐review and editing‐equal.

## RELATED WIREs ARTICLES

https://doi.org/https://doi.org/10.1002/wdev.240

https://doi.org/https://doi.org/10.1002/wdev.91
